# Apoptotic Receptors and CD107a Expression by NK Cells in an Interaction Model with Trophoblast Cells

**DOI:** 10.3390/cimb46080528

**Published:** 2024-08-16

**Authors:** Valentina A. Mikhailova, Dmitry I. Sokolov, Polina V. Grebenkina, Dmitry O. Bazhenov, Igor P. Nikolaenkov, Igor Yu. Kogan, Areg A. Totolian

**Affiliations:** 1 FSBSI “The Research Institute of Obstetrics Gynecology and Reproductology Named after D.O.Ott”, 199034 Saint-Petersburg, Russia; falcojugger@yandex.ru (D.I.S.); ikogan@mail.ru (I.Y.K.); 2 Saint-Petersburg Pasteur Institute, 197101 Saint-Petersburg, Russia; totolian@spbraaci.ru

**Keywords:** NK cells, death receptors, decoy receptors, TRAIL, Fas, FasL, trophoblast, chorionic villi, pregnancy

## Abstract

Natural killer cells (NK cells) exert cytotoxicity towards target cells in several ways, including the expression of apoptosis-mediating ligands (TRAIL, FasL). In addition, NK cells themselves may be susceptible to apoptosis due to the expression of TRAIL receptors. These receptors include TRAIL-R1 (DR4), TRAIL-R2 (DR5), capable of inducing apoptosis, and TRAIL-R3 (DcR1), TRAIL-R4 (DcR2), the so-called “decoy receptors”, which lack an intracellular domain initiating activation of caspases. Of particular interest is the interaction of uterine NK cells with cells of fetal origin, trophoblasts, which are potential targets for natural killer cells to carry out cytotoxicity. The aim of this work was to evaluate the expression of proapoptotic receptors and their ligands as well as CD107a expression by NK cells in a model of interaction with trophoblast cells. To evaluate NK cells, we used cells of the NK-92 line; cells of the JEG-3 line were used as target cells. The cytokines IL-1β, IL-15, IL-18, TNFα, IL-10, TGFβ and conditioned media (CM) of the first and third trimester chorionic villi explants were used as inducers. We established that cytokines changed the expression of apoptotic receptors by NK cells: in the presence of TNFα, the amount and intensity of Fas expression increased, while in the presence of TGFβ, the amount and intensity of expression of the DR5 receptor decreased. Soluble chorionic villi factors alter the expression of TRAIL and FasL by NK-92 cells, which can reflect the suppression of the TRAIL-dependent mechanism of apoptosis in the first trimester and stimulating the Fas-dependent mechanism in the third trimester. In the presence of trophoblast cells, the expression of TRAIL and DcR1 by NK cells was reduced compared to intact cells, indicating an inhibitory effect of trophoblast cells on NK cell cytotoxicity. In the presence of chorionic villi CM and trophoblast cells, a reduced number of NK-92 cells expressing DR4 and DR5 was found. Therefore, soluble factors secreted by chorionic villi cells regulate the resistance of NK cells to death by binding TRAIL, likely maintaining their activity at a certain level in case of contact with trophoblast cells.

## 1. Introduction

Natural killer cells (NK cells) are innate lymphoid cells that can exert cytotoxicity towards target cells in several ways [1]. They are able to release cytotoxic activity via similar mechanisms as T cells, yet NK cells do not require peptide-MHC complex recognition for activation [2].

The NK cell mechanism of cytotoxicity by the release of lytic granules is reflected in the appearance of the protein LAMP1 (CD107a), part of the lysosome membranes, on the NK cell plasma membrane [3,4]. CD107a also determines perforin trafficking into lytic granules [3]. In cases of impaired CD107a expression, there is no delivery of granzyme B into target cells, while the ability of cells to form immunological synapses remains unchanged [3].

In addition to transferring granzymes into the target cell, NK cells can implement a receptor-mediated mechanism of apoptosis [1]. These receptors include receptors for TRAIL—TRAIL-R1, TRAIL-R2, capable of inducing apoptosis of the target cell, and TRAIL-R3, TRAIL-R4, the so-called “decoy receptors”, which lack an intracellular domain that initiates the transmission of a proapoptotic signal and subsequent activation of caspases [5,6]. The TRAIL-R1 and TRAIL-R2 receptors are often referred to as DR4 and DR5 (for death receptor), respectively. TRAIL-R3 and TRAIL-R4 are designated DcR1 and DcR2 (for decoy receptor). Due to the expression of TRAIL, NK cells can cause the death of target cells [6]. In addition, binding of TRAIL causes the production of IFNγ by NK cells, but does not affect their adhesion to target cells [7]. Soluble and cellular microenvironmental factors can influence TRAIL expression density. For example, the expression of TRAIL by NK cells, as well as the cytotoxicity receptors NKG2D and DNAM-1, increased in the case of culturing NK cells on the feeder cell layer [8]. NK cells themselves can express receptors for TRAIL that was shown for peripheral blood NK cell expression of DR5 and DcR1 [9].

The receptor-mediated mechanism for apoptosis induction by NK cells is also implemented by FasL proteins. The localization of these proteins in membrane vesicles that are part of cytolytic granules has been shown [10]. After the formation of an immunological synapse, some of these vesicles move to the contact area with the target cell and degranulate [10]. It has been described that binding of FasL to Fas on the target cell leads to the activation of a proapototic cascade, leading to the activation of caspases and depolarization of mitochondrial membranes [4].

Of particular interest is the interaction of uterine NK cells with cells of fetal origin, trophoblasts, which are potential targets for natural killer cells to carry out cytotoxicity. Studies of uterine NK cells and trophoblast cells are often carried out using animal models and cell lines. It was previously established that mouse trophoblast cells, as they differentiate, increased the secretion of the soluble form of TRAIL [11]. It has also been demonstrated that the interaction of peripheral blood NK cells with trophoblast cells of the JEG-3 line leads to the death of trophoblast cells [12]. The aim of this work was to evaluate the expression of proapoptotic receptors and their ligands as well as CD107a expression by NK cells in a model of interaction with trophoblast cells.

## 2. Materials and Methods

### 2.1. Cell Lines

We used cells of the NK-92 line to evaluate NK cells, for which, according to ATCC (Manassas, VA, USA), the main characteristics of NK cells were described [13,14,15,16,17]. Cells of the JEG-3 line were used as target cells for the cytotoxic activity assessment; their morphology and phenotypic characteristics corresponded to extravillous invasive trophoblast cells [15,18,19,20,21,22,23]. Before experiments, cells were cultured at 5%CO_2_ 37 °C according to the protocols recommended by ATCC (USA). Briefly, for NK-92 cells, we used αMEM based culture medium supplied by Donor Horse Serum, Fetal Bovine Serum, human recombinant IL-2 (Merck, Rahway, NJ, USA), for JEG-3 cells DMEM based culture medium was used containing Fetal Bovine Serum (Merck, USA).

### 2.2. Inductors

The cytokines used in the study were IL-1β (Betaleukin, Saint-Petersburg, Russia) 100 U/mL, IL-15 (R&D, Minneapolis, MN, USA) 10 ng/mL, IL-18 (R&D) 10 ng/mL, TNFα (Refnolin, Vilnius, Lithuania) 50 U/mL, IL-10 (R&D, USA) 10 ng/mL, TGFβ (R&D, USA) 5 ng/mL. The secretion of these cytokines by uterine and placental cells was previously shown [24,25], including endometrial cells [26], trophoblast cell [27,28,29], macrophages [30,31,32], decidual stromal cells [33].

### 2.3. Conditioned Media (CM) of Chorionic Villi Explants

Chorionic villi were obtained after induced abortion in the first trimester of normal pregnancy in somatically healthy women without a history of reproductive disorders (n = 20) or after cesarean section in the third trimester of normal pregnancy (n = 15). Indications for surgical delivery included diseases not associated with obstetric pathology, such as high myopia, degenerative and dystrophic diseases of the spine, and cicatricial deformity of the cervix. Placental tissue explants were obtained from the central part of the placenta. After chorionic villi were isolated, we incubated them in DMEM medium (1 mL) without the addition of Fetal Bovine Serum for 24 h. After cultivation, we assessed the average of chorionic villi explant weight, it was 110 ± 15 mg. Conditioned media (CM) of chorionic villi were collected and frozen. Before experiments, we used Bradford method to assess the total amount of protein in the samples with the spectrophotometer NanoDrop One (Thermo Scientific, Waltham, MA, USA). Then chorionic villi CM, which total protein content was 1 mg/mL, were added to the wells. All experiments with CM of chorionic villi of the first and third trimesters were carried out with three repetitions for each combination of cells and CM.

The study was carried out in accordance with the Code of Medical Ethics of the World Medical Association (Declaration of Helsinki). The study plan was approved by the Local Ethics Committee of the Federal State Budgetary Scientific Institution “The Research Institute of Obstetrics, Gynecology and Reproductology named after D.O. Ott” (protocol No. 107 dated 15 March 2021). Informed consent for the examination was obtained from the patients included in the study.

The authors express their gratitude to Ph.D. Belikova M.E. for assistance in obtaining clinical material after induced abortion, used as a source of chorionic villi in the first trimester of pregnancy.

### 2.4. Determination of NK-92 Cell Expression of Proapoptotic Receptors after Incubation with JEG-3 Trophoblast Cells and Inducers

The day before the experiment, JEG-3 cells were subcultured at a concentration of 3 × 10^6^ cells in 10 mL of complete DMEM medium. After 24 h, cells were treated with a solution of 4 μM CFSE (Sigma-Aldrich, St. Louis, MO, USA) according to the manufacturer’s recommendations. Then, the monolayer of CFSE-treated trophoblast cells was disintegrated using a mixture of versene and trypsin solutions (3:1) (Biolot, Russia (BD, Franklin Lakes, NJ, USA)). JEG-3 cells were added to the wells of a round-bottomed 96-well plate. The concentration of trophoblast cells was maintained as 30,000 cells in 50 μL of complete DMEM medium. The NK-92 cell concentration was 150,000 cells in 100 µL. The resulted effector:target ratio was 5:1. After this, we added IL-2 (for 500 UI/mL concentration) to each well. Cytokines in various concentrations or chorionic villi CM were added to the resulting mixture. We used the following concentrations of cytokines: IL-1β (100 U/mL), IL-15 (10 ng/mL), IL-18 (10 ng/mL), TNFα (50 U/mL), IL-10 (10 ng/mL), TGFβ (5 ng/mL) or chorionic villi CM in the first and third trimesters. The cells were centrifuged at 100× *g* for 3 min, and incubated for 4 h (37 °C, 5%CO_2_). The part of cells was cultured without trophoblast cells, in monoculture with and without cytokines, to serve as an internal control for coculture of NK-92 cells and JEG-3 cells. Then the cells were treated with antibodies to CD45 (PerCP), TRAIL (PE), DR4 (PE), DR5 (APC), DcR1 (APC), CD107a (AF700), Fas (FITC), and FasL (APC), combined into three panels of antibodies in order to assess receptors labeled with the same fluorochrome, according to the manufacturer’s recommendations (BD, USA). CD45 was used to distinguish NK-92 cells from JEG-3 trophoblast cells. To control the cytotoxicity reaction, cells of the NK-92 and JEG-3 lines were additionally cultured without inducers. Then the mixture of cells was treated with a solution of propidium iodide (1 mg/mL, Sigma-Aldrich, Merck, Germany). Cytokine experiments were performed in triplicate with three repeats for each combination of cells and cytokines (n = 3). Experiments with CM of chorionic villi of the first and third trimesters were carried out with three repetitions for each combination of cells with CM. Fluorescence analysis was performed using a FacsCantoII flow cytometer (BD, USA).

### 2.5. Statistical Data Analysis

The obtained data were statistically analyzed with the GraphPad Prism 8 software (GraphPad Software, San Diego, CA, USA). To assess the distribution of sample data, the Shapiro-Wilk test was used. Homogeneity of variances was assessed with Bartlett test. Due to the variances being unequal, we further used non-parametric statistics for data analysis (Mann–Whitney U test and Kruskal–Wallis test followed by post-hoc Dunn’s test).

## 3. Results

### 3.1. Expression of Proapoptotic Receptors and Their Ligands by NK-92 Cells in the Presence of Cytokines

**Figure 1:**
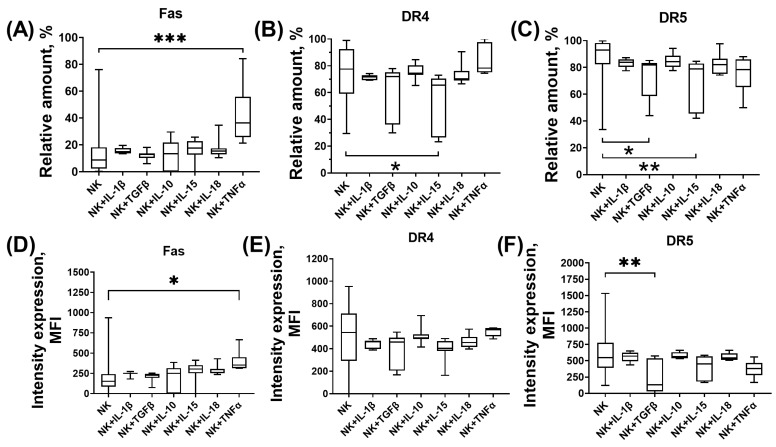
NK-92 cells express receptors and ligands associated with cytotoxic function after incubation with cytokines. Relative number of NK cells expressing Fas (**A**), DR4 (**B**), DR5 (**C**), intensity of expression of Fas (**D**), DR4 (**E**), DR5 (**F**) by NK cells. Statistical significance of differences: * *p* < 0.05, ** *p* < 0.01, *** *p* < 0.001.

In a model of cytotoxic activity against trophoblast cells of the JEG-3 line, changes in the expression of proapoptotic receptors and ligands by NK-92 cells incubated without cytokines and in their presence were assessed. We found that after incubation with TNFα, the number of Fas+ cells of the NK-92 line and the intensity of Fas expression were increased compared to the number of these cells and the intensity of expression without the inducers (Figure 1A,D). The number of NK cells expressing DR4 and DR5 was reduced in the presence of IL-15 compared to the number of these cells in the absence of inducers (Figure 1B,C). The number of DR5+ NK cells and their intensity of DR5 expression were reduced in the presence of TGFβ compared to unstimulated NK cells (Figure 1C,F). Expression of other proapoptotic receptors and ligands by NK cells was not altered in the presence of cytokines.

### 3.2. Expression of Proapoptotic Receptors and Their Ligands by NK-92 Cells in the Presence of Trophoblast Cells and Cytokines

**Figure 2:**
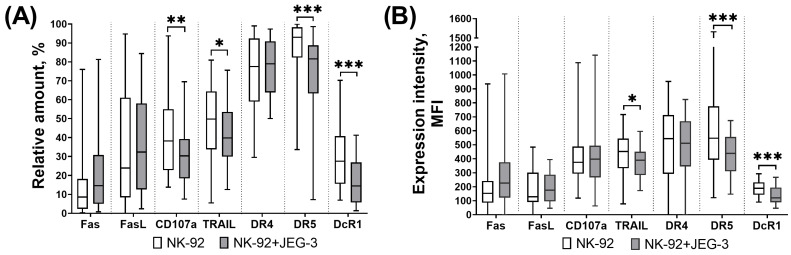
Expression of receptors and ligands associated with cytotoxic function by NK-92 cells in a model of their cytotoxic activity. Relative number of NK-92 cells (**A**), intensity of expression (**B**). Statistical significance of differences: * *p* < 0.05, ** *p* < 0.01, *** *p* < 0.001.

We have previously shown that NK-92 cells cause the death of JEG-3 trophoblast cells [34]. In the presence of JEG-3 trophoblast cells, NK-92 cells change the expression of proapoptotic receptors. The relative number of NK cells expressing the DR5 and DcR1 receptors, as well as the membrane proteins CD107a and TRAIL, was reduced after contact with JEG-3 cells compared to intact NK cells (Figure 2A). Compared to the expression by intact NK cells, the expression intensity of TRAIL, DR5 and DcR1 by NK-92 cells was also reduced after contact with trophoblast cells (Figure 2B).

**Figure 3:**
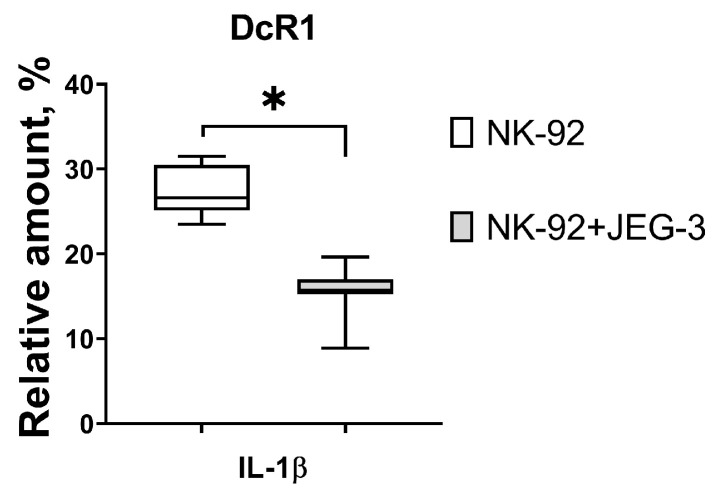
Relative number of DcR1+ cells of the NK-92 line in the model of their cytotoxic activity in the presence of IL-1β. Statistical significance of differences: * *p* < 0.05.

We assessed the effects of separate cytokines on NK cells in the presence of trophoblast cells of JEG-3 cell line to estimate possible leading effects of particular cytokines in coculture. Yet, we showed that only in the presence of IL-1β did the impact of trophoblast cells on NK cell DcR1 expression persist. We detected that JEG-3 trophoblast cells induced a decrease in the number of DcR1+ NK cells compared to NK cells incubated without JEG-3 cells (Figure 3).

In the presence of other cytokines, no differences in the expression of proapoptotic receptors and ligands by NK cells in case of contact interaction with trophoblast cells have been established.

### 3.3. Expression of Proapoptotic Receptors and Their Ligands by NK-92 Cells in the Presence of Chorionic Villi CM

**Figure 4:**
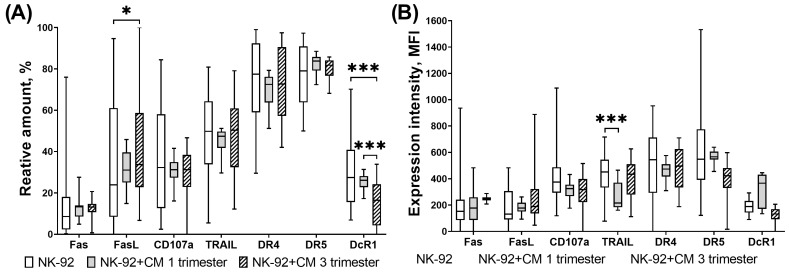
Expression of receptors and ligands associated with cytotoxic function by NK-92 cells after incubation with chorionic villi CM. Relative number of NK-92 cells (**A**), intensity of expression (**B**). Statistical significance of differences: * *p* < 0.05, *** *p* < 0.001.

We found that in the presence of chorionic villi CM of the third trimester of pregnancy, the relative number of FasL+ NK cells was increased, and the number of DcR1+ NK cells was decreased compared to the number of cells incubated without CM (Figure 4A).

The number of NK cells expressing DcR1 was reduced under influence of the third trimester chorionic villi CM compared to the number of these cells in case of the first trimester CM used (Figure 4A). The intensity of NK-92 cell line TRAIL expression was reduced in the presence of first trimester chorionic villi CM compared to the non-activated NK cell expression level (Figure 4B).

### 3.4. Expression of Proapoptotic Receptors and Ligands of NK-92 Cells in the Presence of Trophoblast Cells and Chorionic Villi CM

**Figure 5:**
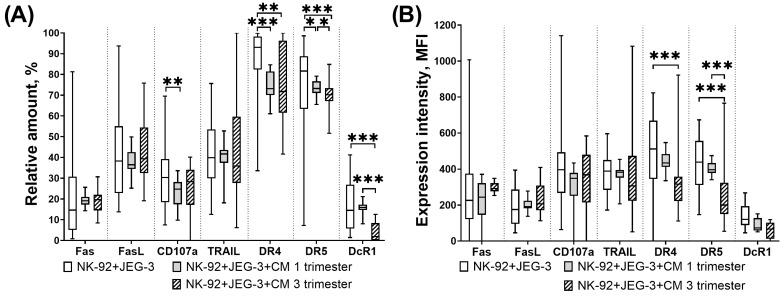
Expression of receptors and ligands associated with cytotoxic function by NK-92 cells in a model of their cytotoxic activity in presence of chorionic villi CM. Relative number of NK-92 cells (**A**), intensity of expression (**B**). Statistical significance of differences: ** *p* < 0.01, *** *p* < 0.001.

We showed that in a model of cytotoxic activity against trophoblast cells of the JEG-3 line, the number of NK-92 cells expressing CD107a, DR4 and DR5 was reduced in the presence of first trimester CM compared to unstimulated cells (Figure 5A).

In case of the third trimester CM comparing to conditions of trophoblast presence without CM, the number of DR4+ DR5+ NK cells as well as the expression intensity of these receptors by NK cells were reduced (Figure 5A,B). The number of DcR1+ NK cells was also reduced in the presence of third trimester chorionic villi CM compared to the number of these cells in the presence of trophoblast but without CM (Figure 5A).

Comparing to the NK cell number in the presence of trophoblast cells and first trimester CM, the amount of DR5+ DcR1+ NK cells declined in the presence of trophoblast cells and third trimester chorionic villi CM (Figure 5A). The expression intensity of DR5 by NK-92 cells was also reduced in the presence of trophoblast cells and third trimester chorionic villi CM, compared to the intensity expression in the presence of trophoblast cells and first trimester chorionic villi CM (Figure 5B).

**Figure 6:**
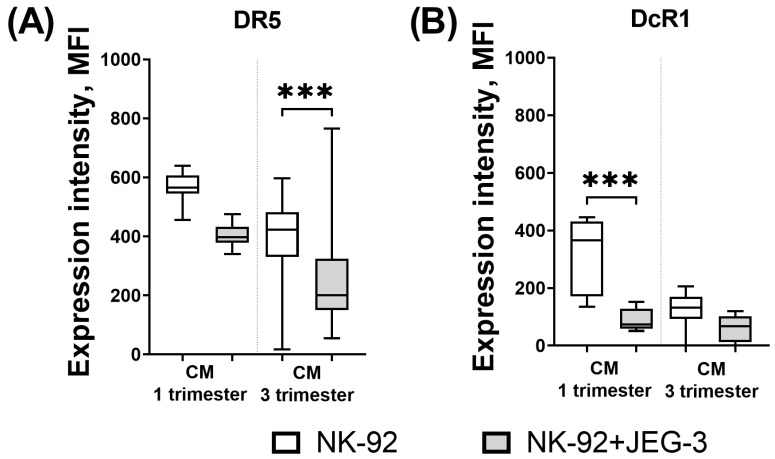
Intensity of expression of DR5 (**A**) and DcR1 (**B**) receptors by NK-92 cells in the model of their cytotoxic activity in the presence of chorionic villi CM. Statistical significance of differences: *** *p* < 0.001.

It is necessary to note the decrease in the expression intensity of the DR5 receptor by NK cells in the presence of third trimester CM and trophoblast cells compared to its expression by NK cells only in the presence of CM. Comparing with the intensity of DcR1 expression only in the presence of first trimester CM, the DcR1 expression by NK cells in the presence of trophoblast cells and first trimester CM was reduced (Figure 6). No other differences in the influence of trophoblast cells on NK cells in the presence of CM were identified.

## 4. Discussion

In this study, we used a model of NK cell cytotoxic activity towards trophoblast cells established with the cellular lines NK-92 and JEG-3. The use of cellular lines in the study instead of decidual (d)NK cells was preferable, as it enabled the assessment of cytokine and placental soluble factors influence under standard conditions. This work demonstrates that NK-92 cells, in addition to FasL, also express Fas, that corresponds to the literature data [35]. Although Fas expression makes cells vulnerable to other NK cells in the microenvironment, it has previously been shown that Fas binding does not lead to a marked increase in the number of dead NK cells: less than 10% of cells expressed annexin V [35]. After Fas binding, NK-92 cells remained capable of implementing cytotoxicity, but to a lesser extent than intact cells [35]. According to the literature, placental cells secrete various cytokines, including TNFα, IL-15, and TGFβ [36,37,38]. We have established the increased number of Fas+ NK cells and the intensity of their expression of Fas in the presence of TNFα. Since Fas expression makes cells potential targets for the cytotoxicity of other NK cells and T lymphocytes, our results may reflect a mechanism of autoregulation of NK cell population numbers under inflammatory conditions. In addition, the Western Blot method demonstrated the content of Fas and FasL in primary extravillous trophoblast cells and the microvesicles they form [39]. In the third trimester placentas, trophoblast cells are characterized by strong expression of both Fas and FasL [40]. It has been described that under pathological conditions, for example, preeclampsia, the formation of FasL+TRAIL+ microvesicles by placental cells is increased [41]. Accordingly, based on the literature data and the results of the study, the regulation of the number of NK cells in the uterus under conditions of inflammation can be carried out due to Fas-FasL interaction with trophoblast cells. In addition, previously it was demonstrated that elevated secretion of TNFα by dNK cells was associated with recurrent pregnancy loss [42]. Decidual macrophages were shown to over-express FasL at spontaneous abortions [43]. As dNK cells are essential for adequate trophoblast invasion [44], it can be assumed that inflammatory conditions can not only influence dNK cell activity, but also have an impact on their viability. Analyzing the amount of NK cells expressing Fas at recurrent pregnancy loss may provide insight into the mechanisms of this pathology.

We have established the expression of TRAIL and its functional receptors DR4 and DR5 by NK-92 cells, which is consistent with literature data [45]. The expression of DR4 and DR5 receptors by NK cells, as well as Fas, probably reflects one of the ways of regulating their numbers. Hofle et al. showed that the expression of CD107a by NK cells correlates with increased expression of TRAIL on the NK cell membrane and the implementation of cytotoxicity by them [7]. It is described that binding of NK cell TRAIL also activates the IL-15 signaling pathway that increases the synthesis of GrzB in NK cells [45]. Accordingly, TRAIL expression by NK cells reflects their activation status and readiness to implement the cytotoxic function.

The cytokine IL-15 is pleiotropic in its effects on NK cells, demonstrating, depending on microenvironmental conditions, both stimulation of the cytotoxic functions of NK cells localized in tumors and stimulation of lymphoproliferative diseases [46]. This study found that IL-15 caused a decrease in the number of DR4+ DR5+ NK cells. According to the literature, after peripheral blood NK cells cultured with IL-15, their cytotoxicity to K562 target cells increases [47]. Yet, in this study there were no changes in DR4 or DR5 expression intensity by NK cells in case of IL-15 supplementation. Accordingly, the decrease in the number of DR4+ DR5+ NK cells may be due to their death after the implementation of cytotoxicity.

We demonstrated that the number of DR5+ NK-92 cells and the intensity of their expression of DR5 were reduced as a result of incubation with TGFβ. This cytokine causes regulatory transformation of NK cells. Nonetheless, in case of prolonged supplementation, TGFβ stimulates NK cells for the proinflammatory cytokines secretion as well as increased cytotoxicity [48,49]. A decrease in DR5 expression may reflect the loss of the autoregulation mechanism in cells and be an early stage of an increase in their cytotoxicity.

The work assessed the expression of proapoptotic receptors by NK-92 cells in the case of in vitro interaction with trophoblast cells. It was found that as a result of incubation with trophoblast cells of the JEG-3 line, the expression of TRAIL, DR5, and DcR1 by NK-92 cells was reduced, as was the number of NK cells expressing these receptors. Previously, Western Blot showed that trophoblast cells contained TRAIL [39]. Laskarin, G. et al. demonstrated that trophoblast cells from the first trimester of pregnancy weakly expressed TRAIL [50]. IHC studies showed TRAIL expression by trophoblast cells at the first and the third trimesters of pregnancy [51,52]. Though the decreased expression of TRAIL during syncytium formation has been described [51]. According to the literature, trophoblast cells of the JEG-3 line contain TRAIL mRNA, as well as its functional receptors DR4, DR5 and the decoy receptor DcR1 [53,54]. Summing up the results of the study and the literature data, we can assume that TRAIL binding is likely both by NK cells and trophoblast cells. The effect of trophoblast cells of the JEG-3 line on the expression of TRAIL and DcR1 by NK cells in the system we used can be associated with the inhibitory effect of trophoblast cells. Thus, trophoblast cells not only avoid NK cell cytotoxicity but also cause greater susceptibility of NK cells themselves to the induction of apoptosis due to inhibition of the protective mechanism of NK cells, DcR1 expression. At the same time, we showed that NK-92 cells retain their effector functions, maintaining the expression of FasL, DR4 and reducing the expression of DR5 in the presence of trophoblast cells.

In this study, we detected a reduced number of CD107a+ cells of the NK-92 line after contact interaction with JEG-3 trophoblast cells, while the intensity of CD107a expression did not change. In general, the interaction of trophoblast cells of the JEG-3 line with NK cells leads to the induction of NK cell cytotoxicity [55]. The detected in the study reduced number of CD107a+ NK cells after contact with JEG-3 cells is probably associated with the depletion of lytic granules. The results obtained highlight the need to directly assess the number of dead target cells during in vitro assessment of the NK cell cytotoxic function.

In the presence of certain cytokines in the medium, the influence of JEG-3 trophoblast cells on NK-92 cells decreased: the expression of TRAIL and DR5 by NK cells did not change, and the expression of the decoy receptor DcR1 was reduced only in the presence of IL-1β. Thus, extrapolating the obtained results, we can assume that most cytokines stimulate NK cells, increasing their cytotoxic potential relating to trophoblast.

Placental cells secrete a spectrum of various cytokines [29,36,37,38,56] that can influence immune cell interaction with fetal cells, and therefore, at the next stage of the study, we assessed the cumulative effect of soluble factors secreted by chorionic villi cells on the expression of proapoptotic receptors by NK-92 cells. It was found that in the presence of first trimester chorionic villi CM, the expression of TRAIL by NK-92 cells was reduced. In the presence of third trimester CM, the number of FasL+ NK-92 cells increased compared to intact cells. In the presence of trophoblast cells, similar changes were not observed. The obtained data suggest the determining influence of the cytokine microenvironment, which in the first trimester probably suppresses TRAIL-dependent, and, in the third trimester, stimulates the Fas-dependent mechanism of induction of apoptosis by NK cells.

In the presence of trophoblast cells, the first trimester CM caused a decrease in the number of DR4+ and DR5+ NK-92 cells. In addition, in the presence of third trimester chorionic villi CM, the influence of JEG-3 trophoblast cells on NK cells remained: their expression of DR5 was reduced. The established differences can be extrapolated to the reduced susceptibility of NK cells to TRAIL-dependent apoptosis under the influence of trophoblast cells and chorionic villi CM in both the first and third trimesters. This is likely due to the fact that NK cell cytotoxicity is integral to placental development.

**Figure 7:**
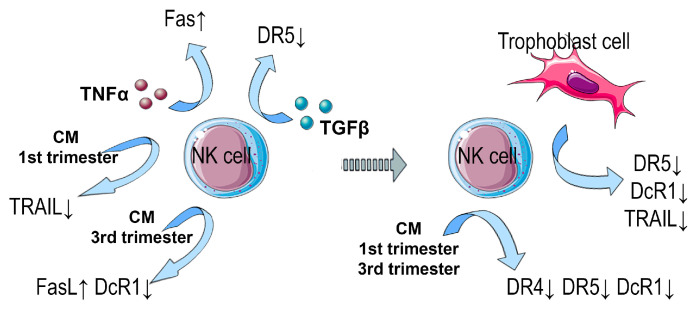
The scheme of NK cell–trophoblast cell interaction via apoptotic receptors based on the main results obtained in the study.

The study shows that in the presence of soluble factors of the first trimester chorionic villi in the medium, trophoblast cells of the JEG-3 line caused a decrease in the intensity of DcR1 expression by NK-92 cells. A decrease in the number of DcR1+ cells of the NK-92 line was established under the influence of the third trimester CM, both in the presence of trophoblast cells and without them. Since DcR1 is a decoy receptor that “protects” NK cells from apoptosis-inducing signals, a decrease in its expression in the model we used can reflect the mechanism of the NK cell population control by trophoblast cells during pregnancy. The main results of the study are summarized in Figure 7.

## 5. Conclusions

Thus, we assessed the pattern of proapoptotic receptor expression by NK cells in a model system using NK-92 and JEG-3 cell lines. We established that microenvironmental cytokines change the expression of apoptotic receptors by NK cells: TNFα stimulated the increase in the amount and expression intensity of Fas, while in the presence of TGFβ, the amount and intensity of expression of the DR5 receptor decreased. Soluble chorionic villous factors alter the expression of TRAIL and FasL by NK-92 cells, which can reflect the suppression of the TRAIL-dependent mechanism of apoptosis induction in the first trimester and stimulating the Fas-dependent mechanism in the third trimester. In the presence of trophoblast cells, NK-92 cells reduced the expression of TRAIL and DcR1 compared to intact cells, indicating an inhibitory effect of trophoblast cells on NK cell cytotoxicity. In the presence of chorionic villi CM and trophoblast cells, we found a reduced number of NK cells expressing DR4 and DR5. Therefore, soluble factors secreted by chorionic villi cells influence the NK cell sensitivity to TRAIL-binding-mediated death, likely maintaining their activity at contact with trophoblast cells. We may assume that the mechanism disturbances of NK cell-trophoblast cell interaction via apoptotic receptors can result in reproductive and pregnancy-associated pathologies.

## Data Availability

Data is contained within the article.
